# A Study on the Uniform Distribution and Counting Method of Raw Cow’s Milk Somatic Cells

**DOI:** 10.3390/mi13122173

**Published:** 2022-12-08

**Authors:** Wei Zhou, Xingyu Li, Wanyun Su, Hongbiao Zheng, Guangxin An, Zhilin Li, Shanshan Li

**Affiliations:** School of Mechanical Engineering, Hebei University of Technology, Tianjin 300401, China

**Keywords:** dairy mastitis, microfluidic chip, image processing, cell counting, standard deviation coefficient

## Abstract

The somatic cell count (SCC) in raw milk is an important basis for determining whether a cow is suffering from mastitis. To address the problem of an uneven distribution of somatic cells due to cell-adherent sedimentation, among other reasons, during milk sampling, which in turn results in unrepresentative somatic cell counting, a method is proposed for obtaining a uniform distribution of somatic cells and improving the counting accuracy based on a nine-cell grid microfluidic chip. Firstly, a simulation was performed to verify the uniformity of the somatic cell distribution within the chip observation cavities. Secondly, a nine-cell grid microfluidic chip was prepared and a negative-pressure injection system integrating staining and stirring was developed to ensure that the somatic cells were uniformly distributed and free from air contamination during the injection process. As well as the structure of the chip, a microscopic imaging system was developed, and the nine chip observation cavities were photographed. Finally, the somatic cells were counted and the uniformity of the somatic cell distribution was verified using image processing. The experimental results show that the standard deviation coefficient of the SCC in each group of nine images was less than 1.61%. The automatic counting accuracy of the system was between 97.07% and 99.47%. This research method lays the foundation for the detection and prevention of mastitis in cows.

## 1. Introduction

The somatic cell count (SCC) of raw cow’s milk is the sum of all cells in each milliliter of milk [1]. When a cow’s lactation system is infected with bacteria, the mammary glands secrete large amounts of leukocytes to fight the bacterial invasion, at which point the number of somatic cells in the cow’s milk increases significantly. Therefore, SCC can be used as a basis for determining whether a cow is suffering from mastitis. Identifying cows with mastitis by testing the SCC, then isolating and treating them as early as possible, can effectively prevent the spread of bacteria in the herd to reduce consequential economic losses.

Many scholars have conducted research in the field of milk somatic cell counting and have developed different counting methods. J.M. et al. [2] detected SCC based on the California Mastitis Test. Giovanna et al. [3] counted cow’s milk cells based on flow cytometry techniques. Sabine et al. [4] used flow cytometry to establish high-resolution differential cell counts in blood and milk. Chengolova et al. [5] used a fluorescent automated cell counter Lactoscan SCC for cow’s milk somatic cell counting, and the correlation between the results and flow cytometry counts was 0.98. Shigenobu et al. [6] used scanning electrochemical microscopy to calculate the number of somatic cells in cow’s milk. Gao et al. [7] developed a somatic cell counting instrument based on a vision measurement algorithm, which verified its suitability by comparing the counting results of the Danish FossilMatic 5000. Aline et al. [8] proposed an automatic milk somatic cell counting method using the fuzzy clustering method and image processing technology, which was consistent with the results obtained using conventional counting methods. Zeng et al. [9] designed a T-junction droplet generator that could quantify and accurately detect the number of milk somatic cells from the length of a single dispersion droplet with a deviation of less than 2.0%. Tania et al. [10] compared eight different machine learning methods for predicting cow udder health based on SCC, all with a prediction accuracy of more than 75%. Zhu et al. [11] used the coaxial probe technique to collect a dielectric spectra of raw milk to quantify the SCC. Gamze et al. [12] performed somatic cell counting on fluorescently labeled raw milk based on a microfluidic platform, achieving an accuracy of more than 80%. P.A. et al. [13] predicted the SCC in goat’s milk used for cheese production based on artificial neural networks.

The counting methods mentioned above have made considerable progress, but the following problems exist in comparison. Some methods that directly measure SCC, such as flow cytometry, use instruments which are costly, bulky and difficult to transport. Therefore, they can only be applied in a laboratory environment. These methods cannot be performed on the ranch site and cannot guarantee the freshness of the cow’s milk. There are some methods that indirectly calculate the overall SCC by calculating the SCC in a small number of samples, such as fluorescence microscopy, image detection, etc. These methods often ignore the problems of cell-adherent sedimentation, etc., caused by cell adhesion and gravity, among other reasons. Therefore, somatic cells are unevenly distributed in the sample solution, resulting in the sample being unrepresentative. The SCC in the sample is calculated to have a significant error between the overall SCC and its true value, resulting in an incorrect judgment on the health of the cow’s udder.

The microfluidics field boasts a wide range of applications for cell counting. Wang et al. [14] designed a microfluidic chip based on two pairs of electrodes to perform cell counting using the Coulter principle. Peng et al. [15] proposed a microfluidic chip integrated with a three-dimensional hydrodynamic focusing system and an on-chip optical system for leukocyte counting. Su et al. [16] integrated a digital immunosensor into a microfluidic chip with an on-chip cytometry chamber, allowing for the accurate counting of individual cells. Zhang et al. [17] developed a microfluidic chip for cell counting based on impedance. Sobahi et al. [18] proposed a low-cost, label-free and real-time cell counting method based on microfluidic chips. Zhao et al. [19] used a photogeneration-based microfluidic system to count red blood cells in blood.

In order to solve the above-mentioned problems in the field of cow’s milk somatic cell counting, this paper proposes a method based on a microfluidic chip to obtain a uniform distribution of cow’s milk somatic cells. The innovations in this article are as follows:

1. A nine-cell grid microfluidic chip was prepared to ensure the uniformity of the distribution of somatic cells in the chip. The averages of the SCCs from the nine observation cavities were taken as the final detection result to improve the counting accuracy so that the final SCC was representative. Image processing algorithms and cell counting algorithms will be made easier with the uniform distribution of somatic cells.
2. A combination of microfluidic technology and image processing algorithms was applied to the field of cow’s milk somatic cell counting, developing a low-cost and portable somatic cell counting system with an automatic counting accuracy of up to 97.07% or more, which could be detached from the laboratory environment for on-the-ranch-site testing of cow’s milk. The system integrated the functions of staining, stirring, sample feeding, microscopic imaging, automatic photo-taking, cell counting, etc. The whole process of testing could be controlled within 1 min from the beginning of staining to the end of counting, which met the requirements for the freshness of cow’s milk.

The remaining part of this paper is structured as follows: Section 2 details the materials and methods used, including the cell distribution uniformity of the simulated chip and the image processing algorithms. Section 3 details the system design and experiments, and discusses the results. Section 4 provides the conclusion.

## 2. Materials and Methods

### 2.1. Simulation of Cell Distribution Uniformity

To make the removed samples more representative, the somatic cells should be evenly distributed within the observation cavity. In this study, we used microfluidic chips instead of traditional glass slides as the observation platform for cow’s milk somatic cells. The somatic cells were unevenly distributed underneath the coverslip due to the edge effect of the glass slide. Somatic cells would accumulate in the center of the glass slide, causing adhesion in the cells. The use of glass slides is very demanding on the operator; air bubbles can be introduced when the coverslip is covered as a result of improper handling. These bubbles affect the observation of somatic cells, and the distribution of somatic cells around the bubbles is not uniform. The microfluidic chip, however, allows a microfluidic pressure pump to be used to inject the solution into the chip, creating a laminar flow within the chip without edge effects, and no bubbles occur during injection. Moreover, the injection operation is simple and can be completed by personnel without professional training.

**Figure 1:**
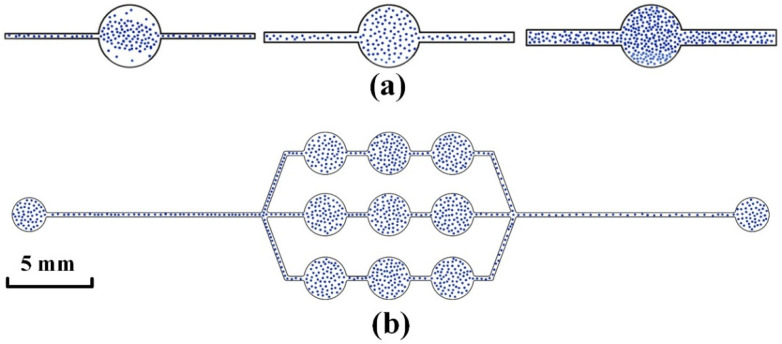
Simulation of cell distribution. (**a**) Particle distribution of observation cavity diameter and flow channel width at different ratios. (**b**) Particle distribution of nine-cell channel.

To improve the counting accuracy, the SCC in one image should not be used as the counting result, but the average of the counting results of nine pictures should be taken as the final SCC. To ensure that nine images of different areas are taken in a single observation cavity, the observation cavity area must be large. Thus, the observation cavity area is too large to ensure the uniformity of somatic cell distribution, as shown in Figure 1a. According to the processability requirements of PDMS, if the observation cavity is too large, this will cause the center of the observation cavity to collapse. The area of the observation cavity is relatively small if a uniform distribution of somatic cells is ensured within a single observation cavity. Taking nine images of a small observation cavity area would result in duplicate images of the same area. Therefore, a nine-cell microfluidic chip structure is proposed [20]. A single image is taken directly above each observation cavity to ensure a uniform distribution of somatic cells while ensuring that the photographed area is not duplicated.

The distribution of milk somatic cells is simulated by the distribution of particles in the observation cavity. Comsol simulation is used to realize the distribution of particles at different ratios of observation cavity diameter and flow channel width. The diameter is fixed during the simulation, while the width is variable. The fluid flow particle tracking physics and laminar flow physics are selected for coupling. The fluid material of the laminar flow field and raw milk have the same density and dynamic viscosity. Where the density is 1039 kg/m^3^, the dynamic viscosity is 0.0017 Pa∙s, and the fluid velocity is 0.02 m/s. The percentage of leukocytes in milk somatic cells is 98%. Therefore, the particles, with the same diameter as the leukocytes, can be chosen instead of leukocytes. Where the diameter is 14 μm, the particle release interval is 0.05 s, and the release time is 1 s. The initial position of the particles is chosen to be evenly distributed, with 50 particles released at a time. The initial velocity of the particles depends on the velocity field of the fluid, and the released particles follow Newton’s second law. The wall condition at the particle exit is set to freeze and the rest of the wall condition is set to bounce. The calculation time is set to 2 s, the results are output every 0.02 s, and the relative tolerance is set to 0.01.

The particle distribution in different ratios of observation cavity diameter and flow channel width is shown in Figure 1a. It is found that if the width is too narrow, the particles will be concentrated in the middle of the observation cavity and will not evenly fill the whole observation cavity. Conversely, a large number of particles will accumulate in the observation cavity, resulting in the phenomenon of multiple particles overlapping. Considering the influence of fluid pressure, fluid velocity, etc., on the channel, the ratio of the diameter of the observation cavity to the width is finally determined to be 14:25, and the particles fill the entire observation cavity uniformly. The particle distribution simulation diagram of the nine-cell grid microfluidic chip calculated by Comsol is shown in Figure 1b. Through the simulation diagram, it can be intuitively seen that the particles are distributed evenly in the nine observation cavities of the microfluidic chip, and thus, the nine-cell grid microfluidic chip can meet the requirements of somatic cell distribution uniformity.

### 2.2. Microfluidic Chip Preparation

**Figure 2:**
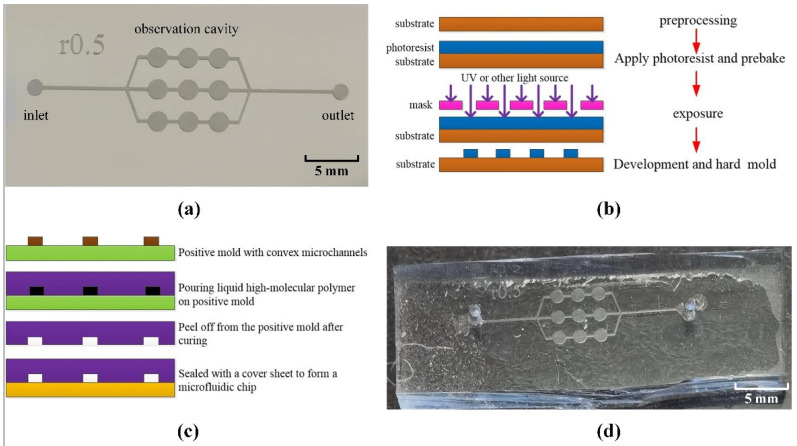
Microfluidic chip preparation. (**a**) Microfluidic chip channel anode mode. (**b**) Photolithography step. (**c**) Microfluidic chip preparation process. (**d**) The nine-cell grid microfluidic chip.

The process of chip preparation can be briefly described as follows. Firstly, an anode with a nine-cell grid microchannel protrusion is produced by photolithography [21], as shown in Figure 2a. The steps of photolithography are shown in Figure 2b. Secondly, the polydimethylsiloxane (PDMS) is cast on the anode [22], and after curing at a certain temperature, PDMS is peeled off from the anode to produce a chip substrate with microchannels. Finally, a polymer microfluidic chip is produced after sealing with a glass cover sheet. Its process is shown in Figure 2c. On the nine-cell grid microfluidic network, there is a set of microfluidic microcavities for loading the sample to be tested, with one end of the microfluidic flow path connected to the inlet and the other connected to the waste cavity through the outlet [23]. In the design, there are three observation cavities evenly distributed in the microfluidic channel, and the whole chip has three channels and nine observation cavities. The microscopic imaging system acquires cell image information directly above every observation cavity. To reduce the aggregation of cells in the observation microcavity and the adhesion in the feed channel, the channel height is designed to be 40 μm. The nine-cell grid microfluidic chip is shown in Figure 2d.

### 2.3. Study on SCC Method Based on Image Processing

The cell counting algorithm is divided into image processing and cell counting. The purpose of image processing is to improve the recognition effect of the image, highlight the target information, reduce the impact of the background information on the target information, and make the processed image more conducive to cell counting. Cell counting involves counting the connected domains in the image after image processing. Image processing is divided into image grayscale, image filtering, image enhancement, Canny edge detection, and morphological opening operation.

**Figure 3:**
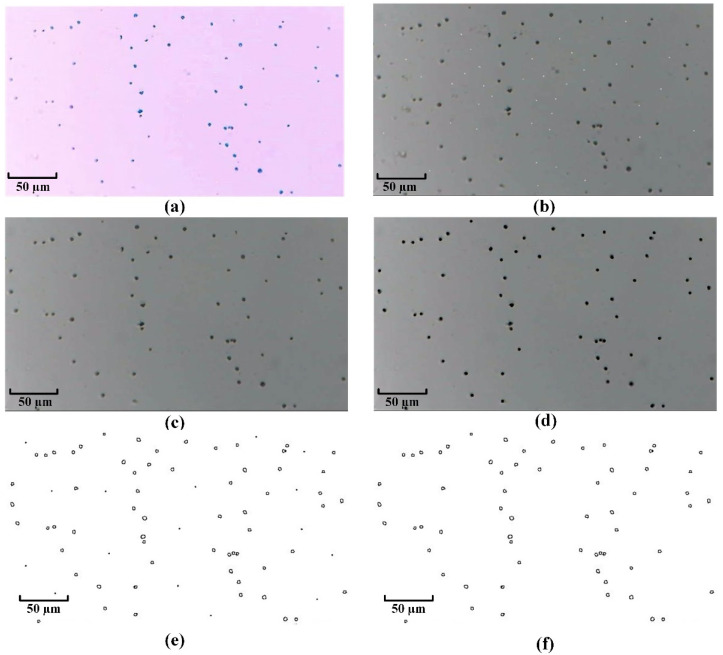
Image processing process. (**a**) Original image. (**b**) Grayscale image. (**c**) Filtered image. (**d**) Enhanced image. (**e**) The image output after the Canny edge detection. (**f**) The final image output after morphological opening operation.

After the cow’s milk cells are stained by Wright’s stain [24], the nuclei appear blue–purple and granular, and the other areas are light pink. The original image (for a clearer illustration of image processing, take 1/9 of the captured image as an example) is shown in Figure 3a. The original photographs taken by the microscopy imaging system are color images, also known as RGB images. Grayscale images occupy less computer memory, faster computation, and facilitate subsequent processing, so the grayscale processing of the image is carried out first.

The weighted value method is selected for grayscale processing of the image [25], as shown in Equation (1).

(1) Gray=0.114Blue+0.587Green+0.299Red 

where red’s weight is 0.299, green’s weight is 0.587, and blue’s weight is 0.114. The grayscale image converted by the weighted value method can not only effectively distinguish the background from the target, but also improve the brightness of the picture without increasing the noise of the picture. The grayscale processed image is shown in Figure 3b.

In the process of shooting, storing, and transmitting photos, it is inevitable that the image will be contaminated by noise due to reasons such as imaging systems or transmission media. Therefore, it is necessary to filter out these impurities to improve the image quality. The images often contain impulse noise or Gaussian noise. Impulse noise can be suppressed using median filtering [26]. The use of bilateral filtering [27] allows filtering Gaussian noise while ensuring that the edge and texture information of the image is not corrupted. The filtered image is shown in Figure 3c, and the overall image becomes very smooth.

When the grayscale image is filtered, this causes a slight image blur while filtering out the noise. First of all, through the matrix’s masking operation [28], the image’s contrast is increased so that the image becomes clearer. Then, the image’s brightness and contrast are further improved by contrast-limited adaptive histogram equalization (CLAHE) [29]. The enhanced image is shown in Figure 3d.

When counting cow’s milk somatic cells, the specific information inside the cells is irrelevant; only the external contours of the cells need to be extracted. In this paper, the edges of the cow’s milk somatic cells in the enhanced image are extracted using the Canny edge detection algorithm [30]. The image output after Canny edge detection is a binary image of the edge, as shown in Figure 3e. Some of the residual noise in the grayscale image is revealed in the edge image. The residual noise in the image is filtered by the morphological opening operation [31], and the final output image is shown in Figure 3f.

The connected domain [32] of an image is the area in an image that has the same pixel value and consists of pixels in adjacent positions. The edges of each cell in the image form a connected domain. By calculating the number of connected domains in the image, the SCC in the image can be obtained.

## 3. Experiments and Discussion of Results

### 3.1. System Design

The system has three main components. The first component is the injection system, which injects the sample to be measured into the microfluidic chip. The second component is the microscopic imaging system, which takes pictures of the cow’s milk somatic cells. The third part is the cow’s milk somatic cell counting software.

**Figure 4:**
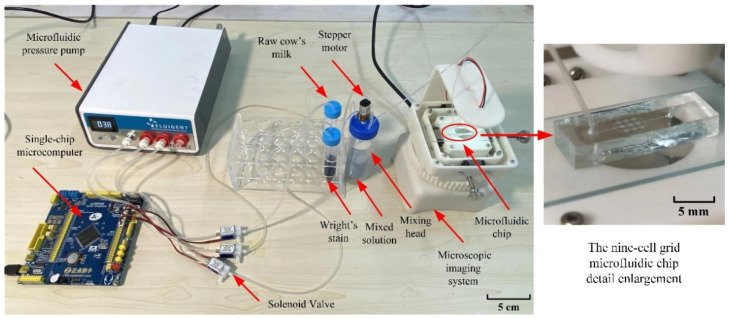
The injection system.

The injection system is divided into three parts, namely, dyeing, stirring, and injection, and is driven by negative pressure to increase the immunity of the system. This prevents the sample from being contaminated with air during the injection process [33], which results in inaccurate counting results. The single-chip microcomputer (STMicroelectronics:STM32F103) is used as the control center of the system and the voltage conversion is realized through the relay. Staining, based on Wright’s staining, is utilized so that raw milk somatic cells appear more clearly in the image. The solenoid valves (JSY:JS0520L) of the reagent bottles, containing the dye and raw milk, are opened and flow into the mixer for mixing. Then, the stepper motor (MOONS’:14H030H-0304) drives the stirring head to stir the mixed solution, evenly distributing the somatic cells in the observation cavity of the microfluidic chip to prevent the problems of cell sedimentation and adherence. Stirring of the mixed solution also allows the raw cow’s milk and the stain to mix well, making the somatic cells more visible in the images. After that, the solution is precisely injected into the microfluidic chip through the microfluidic pressure pump (FLUIGENT: MFCS^TM^ -EZ) [34]. The injection system is shown in Figure 4.

The microscopic imaging system used in this paper is the second version developed. The second version uses 3D printing technology to print the whole body, so that the structure of each part of the microscopic imaging system is integrated. Compared with the first version, the second version is compact, smaller and more portable. The second version of the microscopic imaging system is non-transparent, reducing light interference so that light can only produce diffuse reflection instead of specular reflection, improving imaging quality.

**Figure 5:**
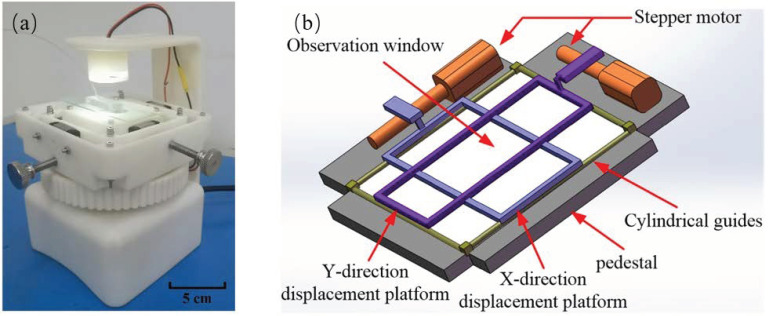
(**a**) Microscopic imaging systems. (**b**) Schematic diagram of the two-degree-of-freedom displacement platform.

The microscopic imaging system consists of two parts: one is a microscopic camera lens, and the other is a two-degree-of-freedom displacement platform, as shown in Figure 5a. The microscopic camera lens can capture 4-megapixel high-definition image information, and the collected image information can be uploaded to the computer for analysis through the WIFI module. The two-degree-of-freedom displacement platform is mainly composed of a base, an X-direction displacement platform, a Y-direction displacement platform, an observation window, a micro-stepper motor, eight circular guides, and other related components. The two-degree-of-freedom displacement stage allows the microscope camera lens to move along the X and Y axes under the control of stepper motors to take an image of each of the nine observation cavities of the microfluidic chip. Figure 5b shows a schematic diagram of a two-degree-of-freedom displacement platform [35]. Through the calculation of the equivalent moment of inertia and equivalent load of the Y stepper motor and the X stepper motor, a micro-stepper motor with a lead screw pitch of 0.5 mm and a moment of inertia of $10^{-9}\;\mathrm{kg}\cdot m^{2}$ is employed. The stepping speed of the two-degree-of-freedom displacement system is 1 mm/s and the rotational speed is 240 r/min.

For the cow’s milk somatic cell counting software, MATLAB was used to write the human–computer interface. The image processing program was written in Visual Studio based on OpenCV and produced directly in MATLAB. The software is divided into three parts: manual counting, automatic counting, and importing databases. When counting manually, each time you click a cell with the mouse, the clicked cell on the image will leave a color marker, and the SCC will increase by 1. Automatic counting is performed on the cow’s milk somatic cells in the images according to the image processing and cell counting algorithms described above. At the end of the automatic count, the software automatically processes the count results and determines the health status of the cow’s udder and the degree of illness. Finally, the software calculates the milking order and treatment order of the cows to be tested. In the importing database section, the software uploads a series of data from the counting process to the SQL database. A health profile of the cow is created in the SQL database [36] for long-term monitoring.

### 3.2. Cell Distribution Uniformity

In this section, 20 cows are randomly selected from local ranches. At the ranch, SCC experiments are performed on the cow’s milk secreted through the cell counting system developed above. The somatic cells are counted in the milk of each cow within one hour of finishing milking to preserve the freshness of the cow’s milk. The nine images of the milk of cow no. 1 are labelled Group 1 images, the nine images of the milk of cow no. 2 are labelled Group 2 images, and so on.

The SCC per image of cow’s milk is shown in Table 1. The SCC standard deviation coefficients for each group of images were calculated, as shown in the penultimate column of Table 1. The average SCC for each group image is different. It is not appropriate to compare the population at different levels directly with the standard deviation indicator. Therefore, the standard deviation coefficient is used to measure the degree of discretization of the SCC in each group of images. The smaller the standard deviation coefficient, the more evenly distributed the somatic cells. As can be seen from the data in the penultimate column of Table 1, the maximum standard deviation coefficient for the SCC of each group of images is 1.61%, the minimum value is 0.21%, and the average value is 0.69%. It is considered that the SCC in the nine observation cavities of the chip fluctuates within the permissible range, which proves that the somatic cells are evenly distributed in the nine observation cavities of the microfluidic chip.

Each image taken through the microscopy imaging system is segmented into an average of 9. At this point, the number of images in each group is 81. The standard deviation coefficients of the SCC for 81 (1/9) images in each group are shown in the last column of Table 1. The uniformity of the distribution of somatic cells within each observation cavity of the microfluidic chip is verified by somatic cell counting, performed for 1/9 images. From the data in the last column of Table 1, it can be seen that the maximum value of the standard deviation coefficient for the SCC of each group of 81 segmented images is 5.85%, the minimum value is 1.03%, and the average value is 2.73%. The SCC in the 81 images after segmentation is considered to fluctuate within the permissible range, demonstrating that somatic cells are evenly distributed within each observation cavity of the microfluidic chip.

### 3.3. System Count Accuracy and Cow’s Udder Health Status

Take the average value of the automatic count of somatic cells in each group of nine original images as the automatic measured value. Take the average value of the manual count of somatic cells in each group of nine original images as the manually measured value. According to the parameters of the image, the length of the image is 5802 μm, the width is 4050 μm, the height of the channel is 40 μm, and the volume of the solution within the field of view is shown in Equation (2).

(2) $$V=5802\times 4050\times 40=9.39924\times 10^{8}\;\mu m^{3}=0.94\;mm^{3}=0.94\;\mu L\;$$

**Figure 6:**
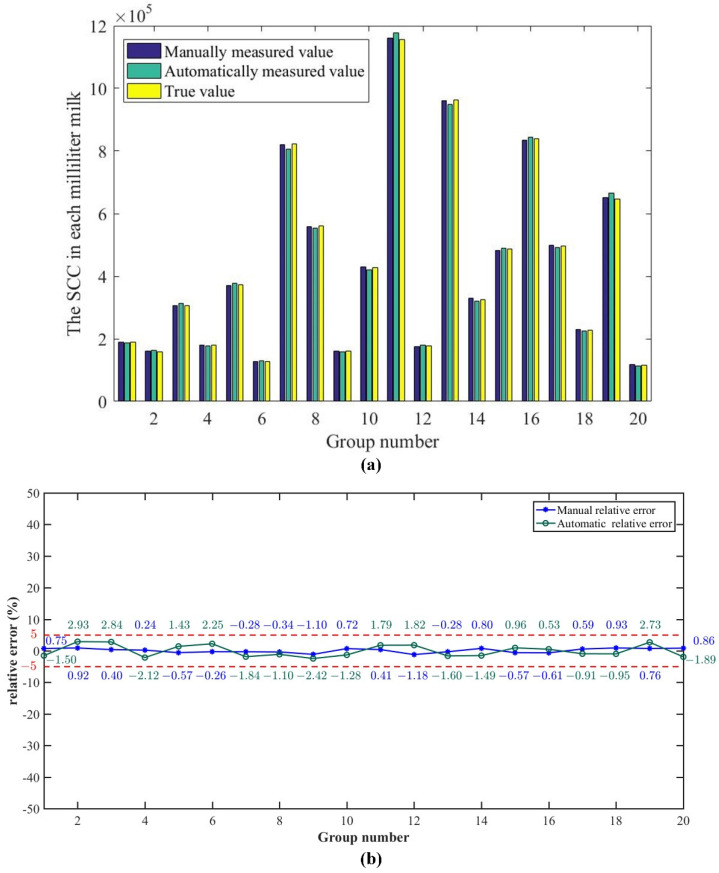
Comparison of the true values with the measured values and error distribution graph. (**a**) The bar graph of measured values compared to true values. (**b**) The error distribution between the measured and true values.

The volume of cow’s milk within the field of view can be derived from Equation (2) as 0.94 μL. The SCC in each milliliter of raw cow’s milk can be derived from the average of the SCC per group of images. BD FACSAria^TM^ flow cytometer counts were used as true values to calculate the accuracy of the manual and automated counts. The comparison between the manually measured values, automatically measured values, and true values of the SCC of each milliliter of raw cow’s milk is shown in Figure 6a. It can be seen that both the manually measured and automatically measured values are close to the true values. The error distribution between the measured and true values is shown in Figure 6b.

As can be seen from Figure 6b, the maximum absolute value of the relative error of the system of automatic counting is 2.93%, the minimum value is 0.53%, and the average value is 1.72%. The maximum relative count error of the automatic counting is obtained as ±2.93%. The maximum absolute value of the relative error of manual counting is 1.18%, the minimum value is 0.24%, and the average value is 0.63%. The maximum relative count error of the manual counting is obtained as ±1.18%. It can be considered that the automatic counting relative error and manual counting relative error are small and within the permissible range. The accuracy rate of the system’s automatic counting is between 97.07% and 99.47%. The accuracy rate of manual counting is between 98.82% and 99.76%. The system has a very high accuracy rate for both automatic and manual counting.

The criteria for judging the health of a cow’s udder by the SCC in cow’s milk are not the same in different studies [37,38,39,40,41]. Combining various factors, this article derives the criteria for judging a cow’s udder health status and the degree of illness of a cow, as shown in Table A1 in Appendix A. The results of judging the measured and true values are shown in Table A2 in Appendix A.

The data in Table A2 in Appendix A show consistency across the results of automatic, manual, and true values for determining a cow’s udder health status. It is verified that the measured values of the automatic counting system are valid, and the judgment results are accurate.

When milking a herd, cows suspected of having subclinical mastitis should be at the end of the herd. If available, they could be equipped with separate milkers so as not to interfere with other healthy cows. Cows already suffering from subclinical mastitis must be isolated and treated to avoid further losses to the ranch. For cow no. 11 already suffering from clinical mastitis, it is recommended that she be taken immediately to a specialized veterinary hospital for systematic treatment.

In summary, the recommended milking order is cow no. 20, cow no. 6, cow no. 9, cow no. 2, cow no. 12, cow no. 4, cow no. 1, cow no. 18, cow no. 3, cow no. 14, cow no. 5, cow no. 10, cow no. 15, and cow no. 17. The recommended order of treatment is cow no. 11, cow no. 13, cow no. 16, cow no. 7, cow no. 19, and cow no. 8.

## 4. Conclusions

In this paper, SCC in raw milk secreted from 20 cows was detected on a ranch with a developed raw cow’s milk somatic cell counting system. The detection results show that the standard deviation coefficient of the SCC in each group of nine images is less than or equal to 1.61%, which verifies the uniformity of the distribution of somatic cells in the nine observation cavities of the microfluidic chip. Each image is evenly divided into 9 images, and the standard deviation coefficient of the SCC in each group of 81 segmented images is less than or equal to 5.85%, which verifies the uniformity of the distribution of somatic cells in each observation cavity of the microfluidic chip. The system ensures the uniform distribution of somatic cells and renders the samples taken more representative. The average value of the SCC in the nine observation cavities was taken as the final detection result and compared with the BD FACSAria^TM^ flow cytometer counting results. It was concluded that the accuracy of the automatic system counts ranged between 97.07% and 99.47%; the accuracy of the manual counts ranged between 98.82% and 99.76%. Because the removed samples are more representative, the judgment of cow’s udder health status is also more accurate. Based on the cow’s udder health status, the cows’ milking order and treatment order are recommended to minimize economic losses to the ranch.

At a later stage, the size of each connected domain in the image can be used to determine the type of somatic cells. Then, the somatic cells can be classified and counted to study the influence of the number of single cells on cow’s udder health. The research and development cost of the raw cow’s milk somatic cell counting system is low, and the application prospects are wide. It provides convenience for detecting and preventing cow mastitis and is a reference for designing other types of cell counting systems.

## Figures and Tables

**Table 1:** Number of somatic cells per image in each group and the standard deviation coefficient.

Group Number	1	2	3	4	5	6	7	8	9	Standard Deviation Coefficient	Standard Deviation Coefficient (1/9)
1	179	180	177	181	182	178	177	182	180	1.08%	4.31%
2	152	151	153	149	150	149	151	152	148	1.11%	4.37%
3	286	290	291	287	287	288	290	287	288	0.60%	2.06%
4	171	169	167	172	173	168	170	172	169	1.19%	3.81%
5	347	350	351	350	349	347	348	347	350	0.45%	1.67%
6	121	118	119	120	117	119	118	121	120	1.17%	5.85%
7	773	770	768	769	771	770	772	768	770	0.22%	1.75%
8	528	525	526	523	525	524	529	526	527	0.36%	1.21%
9	153	152	149	148	151	152	149	150	148	1.24%	4.36%
10	407	403	405	404	406	402	403	407	405	0.45%	1.55%
11	1094	1092	1095	1088	1089	1090	1092	1091	1093	0.21%	1.53%
12	163	167	165	164	163	164	166	165	164	0.81%	3.92%
13	903	905	899	902	907	900	902	905	901	0.29%	1.37%
14	309	312	310	307	308	306	311	312	310	0.69%	2.05%
15	455	456	451	456	457	453	452	451	454	0.50%	2.13%
16	789	783	786	784	782	785	783	780	781	0.35%	1.46%
17	471	470	469	470	468	472	466	471	467	0.43%	1.34%
18	216	214	216	213	215	214	212	216	215	0.66%	3.09%
19	614	613	615	610	616	612	613	611	616	0.35%	1.03%
20	107	109	111	112	109	111	108	110	112	1.61%	5.81%

## Data Availability

The data that support the findings of this study are available from the corresponding author, upon reasonable request.

## Appendix A

**Table A1:** Criteria for judging the cow’s udder health status and degree of illness.

SCC/mL	Cow’s Udder Status	Degree of Illness
SCC < 200,000 /mL	Healthy	
200,000/mL < SCC < 300,000/mL	Subclinical suspicion	Slight
300,000/mL < SCC < 400,000/mL	Subclinical suspicion	Moderate
400,000/mL < SCC < 500,000/mL	Subclinical suspicion	Severe
500,000/mL < SCC < 600,000/mL	Subclinical mastitis	Slight
600,000/mL < SCC < 800,000/mL	Subclinical mastitis	Moderate
800,000/mL < SCC < 1,000,000/mL	Subclinical mastitis	Severe
1,000,000/mL < SCC < 1,200,000/mL	Clinical mastitis	Slight
1,200,000/mL < SCC < 1,500,000/mL	Clinical mastitis	Moderate
SCC > 1,500,000/mL	Severe clinical mastitis	

**Table A2:** The relative error and cow’s udder health status.

Group Number	Manually Measured Value (/mL)	Automatically Measured Value (/mL)	True Value (/mL)	Manual Relative Error	Automatic Relative Error	Cow’s Udder Status	Degree of Illness	Determine Whether the Results Are Consistent
1	190,426	186,170	189,031	0.75%	−1.50%	Healthy		Yes
2	159,574	162,766	158,126	0.92%	2.93%	Healthy		Yes
3	306,383	313,830	305,152	0.40%	2.84%	Subclinical suspicion	Moderate	Yes
4	180,851	176,596	180,413	0.24%	−2.12%	Healthy		Yes
5	370,213	377,660	372,329	−0.57%	1.43%	Subclinical suspicion	Moderate	Yes
6	126,596	129,787	126,931	−0.26%	2.25%	Healthy		Yes
7	819,149	806,383	821,459	−0.28%	−1.84%	Subclinical	Severe	Yes
8	558,511	554,255	560,396	−0.34%	−1.10%	Subclinical	Slight	Yes
9	159,574	157,447	161,345	−1.10%	−2.42%	Healthy		Yes
10	429,787	421,277	426,732	0.72%	−1.28%	Subclinical suspicion	Severe	Yes
11	1,161,702	1,177,660	1,156,930	0.41%	1.79%	Clinical	Slight	Yes
12	175,532	180,851	177,624	−1.18%	1.82%	Healthy		Yes
13	960,638	947,872	963,326	−0.28%	−1.60%	Subclinical	Severe	Yes
14	328,723	321,277	326,129	0.80%	−1.49%	Subclinical suspicion	Moderate	Yes
15	482,979	490,426	485,745	−0.57%	0.96%	Subclinical suspicion	Severe	Yes
16	834,043	843,617	839,188	−0.61%	0.53%	Subclinical	Severe	Yes
17	498,936	491,489	496,021	0.59%	−0.91%	Subclinical suspicion	Severe	Yes
18	228,723	224,468	226,619	0.93%	−0.95%	Subclinical suspicion	Slight	Yes
19	652,128	664,894	647,174	0.76%	2.73%	Subclinical	Moderate	Yes
20	117,021	113,830	116,018	0.86%	−1.89%	Healthy		Yes
